# The Role of Proton Pump Inhibitors in the Management of Pediatric Eosinophilic Esophagitis

**DOI:** 10.3389/fped.2018.00119

**Published:** 2018-05-08

**Authors:** Carolina Gutiérrez-Junquera, Sonia Fernández-Fernández, M. Luz Cilleruelo, Ana Rayo, Enriqueta Román

**Affiliations:** ^1^Pediatric Gastroenterology Unit, Hospital Universitario Puerta de Hierro Majadahonda, Madrid, Spain; ^2^Pediatric Gastroenterology Unit, Hospital Universitario Severo Ochoa, Madrid, Spain

**Keywords:** eosinophilic esophagitis, proton pump inhibitors, esophageal eosinophilia, gastroesophageal reflux disease, pediatrics

## Abstract

Eosinophilic esophagitis (EoE) is a chronic, local, immune-mediated disorder characterized by symptoms of esophageal dysfunction and the presence of a dense eosinophilic infiltrate in the esophageal mucosa. Consensus diagnostic recommendations for EoE diagnosis included absence of histological response to a proton-pump inhibitor (PPI) trial, to exclude gastro-oesophageal reflux disease (GERD)-associated esophagitis. This recommendation exposed an entity known as “proton pump inhibitor-responsive esophageal eosinophilia” (PPI-REE), which refers to patients with EoE phenotype who are PPI-responsive and do not present GERD. In recent years, there is evidence which indicates that PPI-REE is a sub-phenotype of EoE with similar clinical, endoscopic, histological and genetic characteristics, as well as Th2-related inflammatory response. As a result, PPIs should be considered another treatment for EoE and not a diagnostic tool. PPI-REE was originally described in a case series which included two children and in two retrospective pediatric series. Later, a prospective pediatric study showed a high rate of response to PPIs at high doses with long-term maintenance at lower doses. PPI monotherapy in children with esophageal eosinophilia (EE) has been observed to reduce eotaxin-3 expression in epithelial cells and to practically reverse the allergy and inflammatory transcriptome. These data reveal that PPIs are also an effective treatment for EoE in pediatric patients, although more studies are necessary in order to define the best induction and maintenance treatment regimen, the long-term safety profile and their influence on the occurrence of fibrosis and esophageal remodeling.

## Introduction

Eosinophilic esophagitis (EoE) is a chronic, immunologically mediated disorder characterized by symptoms of esophageal dysfunction and the presence of a dense eosinophilic infiltrate in the esophageal mucosa. Its prevalence and incidence have considerably increased in the last few decades and it is currently the most frequent cause of dysphagia and food impaction in children, causing a considerable impact on quality of life (1).

In order to diagnose EoE, it is necessary to exclude other causes of esophageal eosinophilia, such as gastroesophageal reflux disease (GERD). GERD is caused by the exposure of the esophageal mucosa to gastric contents, principally acid, and it is mainly treated with proton pump inhibitors (PPIs). Distinguishing between these two disorders is not always easy in children, where the symptoms of both can overlap and the endoscopic findings of EoE can be more subtle.

Over the last few years, a group of patients with clinical, endoscopic, and histological findings characteristic of EoE and clinical-histological response to PPIs, with normal esophageal pH monitoring results, have been identified. This new disorder was called “PPI-responsive esophageal eosinophilia” (PPI-REE). Recent and evolving evidence have shown that PPI-REE is indistinguishable from EoE, and very different from GERD (2). The objective of this study is to review available scientific evidence regarding the role of PPIs in pediatric EoE.

## Historical perspective

**Figure 1:**
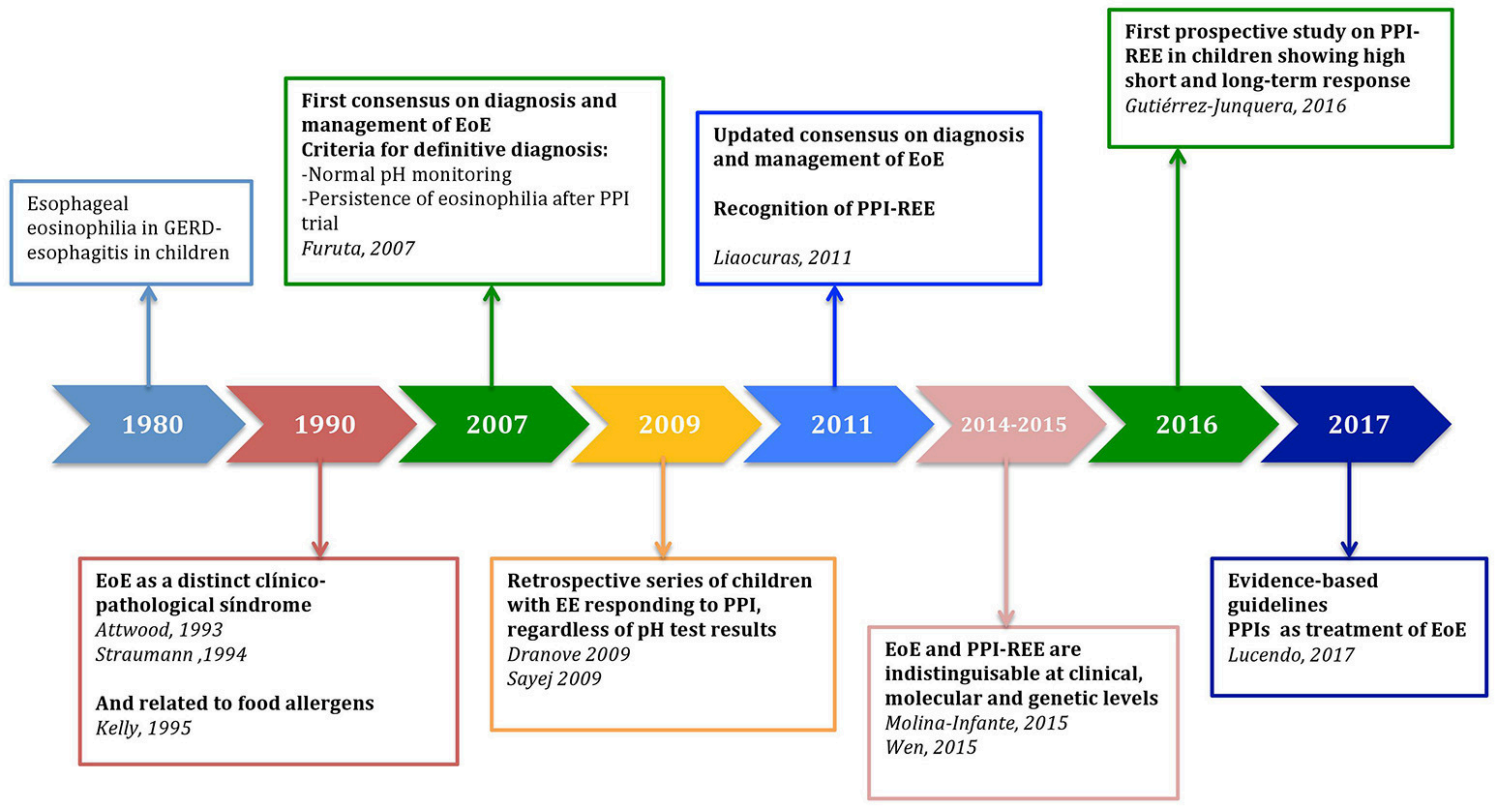
Historical perspective of the role of proton-pump inhibitors in the management of Pediatric Eosinophilic Esophagitis. EoE, eosinophilic esophagitis; EE, esophageal eosinophilia; PPI, Proton-pump inhibitor; PPI-REE, Proton-pump inhibitor responsive esophageal eosinophilia; GERD, gastroesophageal reflux disease.

In the 1980s, the presence of intraepithelial eosinophils was recognized as an histological finding of GERD-associated esophagitis in pediatric patients, it could be present in all levels of the esophagus and was related to esophageal acid exposure on pH monitoring (3) (Figure 1).

In 1993, Attwood et al. described a new disorder in 12 adults with dysphagia and dense esophageal eosinophilic infiltration with normal esophageal pH monitoring (4). In 1994, Straumann et al. described 10 adults with dysphagia, with rings and white plaques in the endoscopy and eosinophilic infiltration of the esophagus, coining the term *eosinophilic esophagitis* (5). In 1995, Kelly et al. uncovered the allergic basis of this new disorder in a series of 10 children previously diagnosed with GERD and refractory esophageal eosinophilia who had undergone medical and surgical treatment. After removing allergenic foods and starting an elemental diet, substantial clinical improvement and a remission of esophageal eosinophilia were observed (6). Later studies involving pediatric patients showed that this new disorder was characterized by a history of atopy and normal esophageal pH monitoring (7). In addition, Steiner et al. demonstrated that the density of the eosinophilic infiltration was not related to the reflux index in children who underwent esophageal biopsy and pH monitoring on the same day; those who presented >20 eos/HPF did not present acid gastroesophageal reflux (8).

These publications introduced the concept of *eosinophilic esophagitis* as an emerging disorder, which was different from GERD. In 2007, the first consensus recommendations regarding the diagnosis and management of EoE in children and adults defined the disorder by the presence of (1) symptoms of esophageal dysfunction including food impaction and dysphagia in adults and food intolerance and symptoms of GERD in children; (2) eosinophilic infiltration of ≥15 eos/HPF; and (3) absence of pathologic GERD as evidenced by either a normal pH monitoring study of the distal esophagus or lack of histological response to high-dose PPI treatment (9). The basis of this recommendation was just that only GERD could respond to PPIs.

As EoE was identified with increasing frequency, the relationship between EoE and GERD was found to be more complex. In 2006, Ngo et al. published a case series of two children and one adult with dysphagia, food impaction and vomiting, furrows, and white plaques in the endoscopy and >20 eos/HPF in the esophageal mucosa. After PPI monotherapy, the symptoms resolved and the eosinophilic infiltration of the esophagus disappeared (10). Later, two retrospective pediatric series published in 2009 showed that 40% of the children with esophageal eosinophilia presented a histological response to PPI treatment, regardless of esophageal pH monitoring results (11, 12). In 2011, a prospective study in adults, which systematically evaluated the response to PPI treatment in adults with dysphagia, food impaction, and esophageal eosinophilia, showed that up to 50% of the patients responded to PPI treatment (13).

This new and unexpected disorder, called PPI-responsive esophageal eosinophilia (PPI-REE), was included in the update to the consensus recommendations in 2011 and it was defined by: typical EoE symptoms and histology, without evidence of GERD by endoscopy or esophageal pH monitoring and with clinico-histological response to PPIs (14, 15). PPI-REE was considered a different disorder from EoE, but not necessarily a manifestation of GERD. Persistence of eosinophilic infiltration (≥15 eos/hpf) after high-dose PPI treatment over a 2-month period was required in order to definitively diagnose EoE.

## PPI-REE prevalence in children

In 2016, a systematic review with a meta-analysis of 33 studies, including 619 symptomatic patients with esophageal eosinophilia (431 adults and 188 children), observed a 50.5% histological remission rate (<15 eos/hpf) after PPI treatment, similar in children and adults, regardless of esophageal pH monitoring results (16), although the quality of the data pertaining to children was low.

The most relevant publications regarding the response to PPI treatment in children with esophageal eosinophilia are summarized in the Table 1. Initial studies were retrospective, with variable PPI doses and treatment durations with a PPI-REE prevalence rate between 22.8 and 60% (11, 12, 17, 18). In 2016, our group presented the first prospective study of response to PPI treatment in 51 children with esophageal dysfunction symptoms and esophageal eosinophilia taking 1 mg/kg/dose of esomeprazole twice daily (19). We observed that 68.6% of the patients had histological remission (<15 eos/HPF) and 47% of them presented complete remission of eosinophilic infiltration (<5 eos/HPF). In our study, the rate of histological remission was higher than in previous studies, which may be related to the implementation of a uniform prospective protocol with high-dose esomeprazole during 8 weeks, and the performance of follow-up endoscopy while on PPI treatment.

**Table 1:** Studies evaluating histological remission rate in pediatric patients with esophageal dysfunction and esophageal eosinophilia.

**Lead author (publication year), type of study**	***n***	**Medication**	**Dose, dosing interval, duration**	**Histological remission rate according to pH monitoring**		**Histological remission rate (definition)**
				**Abnormal pH test results**	**Normal pH test results**	
Dranove et al. (11), Retrospective	43	Omeprazole, esomeprazole or lansoprazole	Not specified	41%	45%.	40% (<5 eos/HPF)
Savej et al. (12), Retrospective	36	Omeprazole, esomeprazole or lansoprazole	1–2 mg/kg/day Twice daily 3 months	Not specified		39% (<15 eos/HPF)
Schroeder et al. (18), Retrospective	35	Omeprazole, esomeprazole	1–2 mg/kg/day Twice daily At least 3 months	Not specified		22.8% (<15 eos/HPF)
Rea et al. (17), Retrospective	25	Not specified	Not specified	69%	44%	60% (not specified)
Gutiérrez Junquera et al. (19), Prospective	51^*^	Esomeprazole	2 mg/kg/day Twice daily 2 months	0 patients out of 1^*^	21 patients out of 30^*^	68.6% (<15 eos/HPF) 47% (<5 eos/HPF)

* *pH testing was performed on 31 of the 57 children. An abnormal pH test result was found in one child, who did not respond to PPI treatment. The remaining 30 children presented normal pH test results and 21 of them responded to treatment. eos/HPF, eosinophils/high-power field*.

In the majority of published studies, the rate of clinical response to PPI treatment was higher than the rate of histological response. In our experience, almost 80% of the children had clinical improvement with PPI treatment regardless of remission of eosinophilic infiltration (19). This emphasizes that symptom resolution may not reflect the objective response to PPI therapy and that a second endoscopy with esophageal biopsies is required to confirm response.

## Predictive factors for PPI-REE in children

In order to avoid repeated endoscopic procedures, many efforts have been made to identify predictive factors of response to PPIs in children with esophageal eosinophilia. However, published studies showed that baseline clinical, histological and endoscopic characteristics were similar in responders and non-responders (11, 19). Patients were indistinguishable in terms of sex, allergy history, and symptoms of esophageal dysfunction. Notably, four of the children included in our study had received food oral immunotherapy treatment for a severe IgE-mediated allergy to milk or egg; three of them responded to PPI treatment, eliminating the need to remove the food from their diet (19). Endoscopic features in PPI responders and non-responders included white plaques, furrows or edema with similar Endoscopic Reference Scores (19). These results concur with those found in studies performed on adults (20–23).

The baseline histological findings were also similar between responders and non-responders, although, in our experience, the mean peak eosinophil count/HPF was higher in non-responders (peak value: 74.8 ± 36.2 in non-responders vs. 46.3 ± 30.7 in responders) (19). Dranove et al. reported that 50% of children who presented between 15 and 20 eos/HPF responded to PPI treatment vs. 29% of those who presented >20 eos/HPF (11). Therefore, it seems that children with higher peak eosinophil counts are less likely to respond to PPIs, although there is not a defined threshold for PPI responsiveness.

Moreover, esophageal pH monitoring did not predict responsiveness to PPI treatment. In our study, pH monitoring was performed on 31 of the 57 children included. Only one child had pathological esophageal acid exposure and did not respond to PPIs (19). In other studies, response to PPIs was observed in 41–69% of the children with pathological esophageal pH monitoring and in 44–45% of children with normal results (11, 17). This fact highlights that response to PPI therapy in children may occur with either normal or pathological esophageal pH monitoring.

Additionally, a case series of patients with EoE, which included a 10-year-old child, showed that patients having responded to an elimination diet or swallowed corticoids may eventually respond to PPI treatment as well, providing further data that PPI-REE is indistinguishable from EoE (24, 25).

## Long-term response to PPI treatment

Initial data suggested that PPI response in children with esophageal eosinophilia was a transient phenomenon and may even correspond to spontaneous fluctuation in the eosinophil counts. Dohil et al. described four patients with initial response to PPI treatment, but subsequent clinical-pathological recurrence while receiving PPI treatment at the same or higher dose over additional 5–17 months; all subjects were reported to be compliant with therapy (26). In a retrospective study, Schroeder et al. reported resolution of esophageal eosinophilia in 7 of 35 patients (20%), two of whom continued on PPI treatment and showed dense eosinophilia in a third endoscopy (18).

However, in our experience, a large proportion of pediatric patients (40 of 57; 70%) with “PPI-REE” remained in clinical-pathological remission on a maintenance dose of 1 mg/kg/day esomeprazole at one year follow-up, or longer, with adequate safety profile (27). We observed that long-term remission rate was higher in children with initial complete histological remission (≤5 eos/hpf) to an 8 week PPI trial than in those with partial remission (>5 and <15 eos/hpf)(81% vs 50%, p = 0.014). Our results corroborate data observed in adults, with a prevalence of sustained histological remission between 73 and 81% in patients with lower-dose PPI maintenance treatment (28, 29). In the study by Molina-Infante et al, 16 patients had stopped PPI treatment; 14 of them had symptom recurrence in the first year after discontinuing PPI therapy, including two who experienced food impaction. The other two patients remained asymptomatic, but histological recurrence was observed in a follow-up endoscopy (29). These data emphasize the concept that PPI-REE is a chronic disease and that clinical-pathological recurrence occurs after treatment discontinuation, as observed in EoE with swallowed steroids.

## Molecular and genetic characteristics of PPI-REE

Novel biomarkers such as genetic targets and measurement of tissue markers and cytokines related to eosinophilic inflammation have also failed to differentiate PPI-REE from EoE. Recent studies in adults have investigated major basic proteins, tryptase staining, and eotaxin-3. Remarkable differentiation of EoE from controls was observed, whereas there was no difference between EoE and PPI-REE (30).

The EoE diagnostic gene panel (EDP), composed of 94 esophageal transcripts, had high sensitivity and specificity for distinguishing pediatric and adult patients with EoE from GERD and control subjects (31, 32). In a multicenter study, a significant genetic overlap between adult and pediatric patients with PPI-REE and those with EoE was observed, including genes for eosinophil chemotaxis (*CCL26*), barrier molecules (desmoglein *DSG1*), tissue remodeling (*POSTN*), and mast cells (*CPA3*) (33). Nevertheless, the study identified a set of candidate genes that may predict PPI responsiveness in EoE patients; a gene encoding potassium channel (KCNJ2/Kir 2.1) (33). More recently, these results have been confirmed in adult patients with a gene expression profile in esophageal mucosa which is indistinguishable between patients with EoE and PRI-REE (34).

In addition, PPI monotherapy is capable of reversing Th2 inflammatory markers in the esophageal mucosa, with a reduction in eotaxin-3 and other Th2-related cytokines levels in adults with PPI-REE (35). In a study performed on children with esophageal eosinophilia, PPI treatment significantly reduced the expression of eotaxin-3 by epithelial cells in the proximal esophagus although not in the distal esophagus (36). Moreover, PPI monotherapy alone almost completely reversed the allergy and inflammatory transcriptome in adult and pediatric patients with PPI-REE (33).

## From “PPI-responsive esophageal eosinophilia” to PPI-responsive EoE

These recent findings indicate that PPI-REE is a sub-phenotype of EoE with similar clinical, molecular and genetic characteristics, as well as an underlying T-helper 2-mediated allergic mechanism. Moreover, PPIs should not be considered a diagnostic tool but a therapeutic option for EoE, as stated by an expert consensus report (2).

The new evidence-based European guidelines for the diagnosis and management of EoE have taken this into account, thus eliminating the need for a trial of PPI treatment in order to make a definitive EoE diagnosis and considering PPI as another treatment (37).

PPI responsiveness has been defined mainly in terms of histological remission. Although the diagnostic threshold for EoE has been established at 15 eos/hpf, there is not uniform criterion to define histological remission in EoE patients after treatment. However, data in adults have shown that a response threshold of 15 eos/hpf is associated with improved symptomatic and endoscopic responses, and that lowering this threshold to less than 15 eos/hpf does not result in a substantial additional symptom or endoscopy response (38).

A validated, parent proxy-reported measure for pediatric EoE have been published (39), but correlation with histological disease activity after treatment has not been established. In adults symptoms do not correlate accurately with histology (40), so esophageal biopsies currently continue to be necessary for monitoring the disease.

## Possible mechanisms of action for PPIS in EoE

How is an esophageal disease mediated by immunoallergic stimulation, such as EoE, able to respond to PPI treatment? Several hypotheses try to explain the mechanism behind PPI response in EoE.

The main effect of PPIs is the inhibition of the H^+^/K^+^ ATPase pump in the parietal gastric cells responsible for acid secretion. After activation in an acid environment, the PPI forms covalent bonds with residual cysteine in the ATPase, rendering the pump inactive. Acid suppression can be beneficial for EoE since acid reflux can damage intercellular connections between epithelial cells, leading to an increase in permeability which, in turn, potentially allows penetration of the mucosa by allergens which cause EoE. In this sense, treatment with high-dose esomeprazole (40 mg, twice daily) improved mucosal integrity, determined by electrical tissue impedance and trans-epithelial electrical resistance, in adult PPI-REE patients (41). Supporting this hypothesis, a series included 3 adult patients with non-PPI responsive EoE that responded to vonoprazan, a novel potassium-competitive acid blocker with a more potent and sustained suppression of gastric acid secretion compared to PPI (42).

However, EoE patients may benefit from PPIs through anti-inflammatory properties, independent from acid suppression. PPIs can inhibit T-helper 2 cytokine-induced eotaxin-3 expression in esophageal epithelial cells in adult patients with EoE, potentially reducing eosinophil recruitment (43). This inhibition was achieved with omeprazole and lansoprazole; in the case of omeprazole, with concentrations as low as 1 mcmol/l, obtainable in the blood through oral or intravenous administration at conventional doses. The inhibitory effect of PPIs seems to involve chromatin remodeling in the eotaxin-3 promoter, resulting in decreased promoter binding of the transcription factor protein, Signal Transducer and Activator of Transcription (STAT) 6 and in reduced eotaxin-3 transcriptional activity in esophageal squamous epithelial cells.

Unlike in epithelial cells, however, omeprazole did not inhibit Th2 cytokine-induced eotaxin-3 expression by esophageal fibroblasts, suggesting that PPIs will have limited impact on sub-epithelial EoE processes such as fibrosis (44).

## PPI-responsive EoE. considerations for pediatric patients

In summary, available data indicate that an important proportion of children with clinical, endoscopic and histological findings characteristic of EoE and normal esophageal pH test results respond to high-dose PPI treatment. PPI monotherapy reduces the expression of eotaxin-3 in esophageal epithelium and practically reverses the allergy and inflammatory transcriptome in children with EoE.

Thus, PPI could be considered as another treatment for pediatric EoE, considering their easy administration and favorable safety profile. Other recommended treatments (swallowed corticoids and elimination diets) are neither universally effective nor free of secondary effects (37). Identifying the food(s) responsible for EoE and eliminating them from the diet probably constitutes the treatment of choice of pediatricians and families due to the absence of adverse effects. However, it is not always possible to identify the allergen or the diet can be very restrictive in children with multiple IgE-mediated food allergies. In other cases, eliminating the allergen can carry a risk of severe IgE-mediated adverse reactions upon reintroduction (45) or due to transgressions, like in the case of children with EoE associated with oral immunotherapy involving food allergens (46).

In recent years, growing concerns have emerged about long-term complications of PPI treatment, but the general long-term safety of these medications in adults is very good (47). No major safety concerns arose during 5–12 years of continuous PPI therapy in a large cohort of adults comparing long-term omeprazole use to anti-reflux surgery (48). Data on the safety of long-term PPI treatment in children are scarce; some studies and case reports indicate a potential association of PPIs with an increased risk of respiratory tract or gastrointestinal infections (49, 50). Due to the potential for adverse events associated with long-term PPI treatment, the lowest effective dose should be used so as to minimize the risk of such events.

Furthermore, PPI response in children with esophageal eosinophilia should not be universally thought of as PPI-responsive EoE. Differentiating between EoE and GERD is more difficult in pediatric patients because the symptoms of EoE are less specific and overlap with those from GERD (heartburn, regurgitation, vomiting, and food refusal) (51). Moreover, endoscopic findings associated with pediatric EoE, generally inflammatory (edema, furrows, and white plaques), can be more subtle. Therefore, when faced with findings such as the presence of unspecific symptoms, macroscopically normal endoscopy, low density eosinophilic infiltration and distal predominance, additional evaluation for GERD is recommended.

The fact that there is less data regarding PPI response in pediatric EoE is partly due to the administration of PPIs prior to the first endoscopy, with the objective of reducing the number of endoscopic and anesthetic procedures. Patients who do not present esophageal eosinophilia are classified as probable cases of GERD. However, faced with evidence of PPI-responsive EoE, this assumption is actually erroneous and generates diagnostic, therapeutic and prognosis uncertainty.

In summary, PPIs are an effective treatment for children with EoE, although more prospective studies are necessary in order to evaluate the best induction and maintenance regimen with regards to dose and duration, as well as the long-term safety profile. Additionally, the effects of PPI treatment on the prevention of fibrosis and esophageal remodeling should be studied.

## Author contributions

All authors listed have made a substantial, direct and intellectual contribution to the work, and approved it for publication.

### Conflict of interest statement

The authors declare that the research was conducted in the absence of any commercial or financial relationships that could be construed as a potential conflict of interest.

## References

[1] Arias Á Pérez-Martínez I Tenías JM Lucendo AJ. Systematic review with meta-analysis: the incidence and prevalence of eosinophilic oesophagitis in children and adults in population-based studies. Aliment Pharmacol Ther. (2016) 43:3–15. 10.1111/apt.13441 26510832

[2] Molina-Infante J Bredenoord AJ Cheng E Dellon ES Furuta GT Gupta SK. Proton pump inhibitor-responsive oesophageal eosinophilia: an entity challenging current diagnostic criteria for eosinophilic oesophagitis. Gut (2016) 65:524–31. 10.1136/gutjnl-2015-310991 26685124 PMC4753110

[3] Winter HS Madara JL Stafford RJ Grand RJ Quinlan JE Goldman H. Intraepithelial eosinophils: a new diagnostic criterion for reflux esophagitis. Gastroenterology (1982) 83:818–23. 7106512

[4] Demeester TR. Esophageal eosinophilia with dysphagia. A distinct clinicopathologic syndrome. Dig Dis Sci. (1993) 38:109–16. 8420741 10.1007/BF01296781

[5] Straumann A Spichtin HP Bernoulli R Loosli J Vögtlin J. [Idiopathic eosinophilic esophagitis: a frequently overlooked disease with typical clinical aspects and discrete endoscopic findings]. Schweiz Med Wochenschr. (1994) 124:1419–29. 7939509

[6] Kelly KJ Lazenby AJ Rowe PC. Eosinophilic esophagitis attributed to gastroesophageal reflux: improvement with an amino acid-based formula. Gastroenterology (1995) 109:1503–12. 7557132 10.1016/0016-5085(95)90637-1

[7] Walsh SV Antonioli DA Goldman H Fox VL Bousvaros A Leichtner AM. Allergic esophagitis in children: a clinicopathological entity. Am J Surg Pathol. (1999) 23:390–6. 10199468 10.1097/00000478-199904000-00003

[8] Steiner SJ Gupta SK Croffie JM Fitzgerald JF. Correlation between number of eosinophils and reflux index on same day esophageal biopsy and 24 hour esophageal pH monitoring. Am J Gastroenterol. (2004) 99:801–5. 10.1111/j.1572-0241.2004.04170.x 15128340

[9] Furuta GT Liacouras CA Collins MH Gupta SK Justinich C Putnam PE. Eosinophilic esophagitis in children and adults: a systematic review and consensus recommendations for diagnosis and treatment. Gastroenterology (2007) 133:1342–63. 10.1053/j.gastro.2007.08.017 17919504

[10] Ngo P Furuta GT Antonioli DA Fox VL. Eosinophils in the esophagus–peptic or allergic eosinophilic esophagitis? Case series of three patients with esophageal eosinophilia. Am J Gastroenterol. (2006) 101:1666–70. 10.1111/j.1572-0241.2006.00562.x 16863575

[11] Dranove JE Horn DS Davis MA Kernek KM Gupta SK. Predictors of response to proton pump inhibitor therapy among children with significant esophageal eosinophilia. J Pediatr. (2009) 154:96–100. 10.1016/j.jpeds.2008.07.042 18783791

[12] Sayej WN Patel R Baker RD Tron E Baker SS. Treatment with high-dose proton pump inhibitors helps distinguish eosinophilic esophagitis from noneosinophilic esophagitis. J Pediatr Gastroenterol Nutr. (2009) 49:393–9. 10.1097/MPG.0b013e31819c4b3e 19633574

[13] Molina-Infante J Ferrando-Lamana L Ripoll C Hernandez-Alonso M Mateos JM Fernandez-Bermejo M. Esophageal eosinophilic infiltration responds to proton pump inhibition in most adults. Clin Gastroenterol Hepatol. (2011) 9:110–7. 10.1016/j.cgh.2010.09.019 20920599

[14] Liacouras CA Furuta GT Hirano I Atkins D Attwood SE Bonis PA. Eosinophilic esophagitis: updated consensus recommendations for children and adults. J Allergy Clin Immunol. (2011) 128:3–20. 10.1016/j.jaci.2011.02.040 21477849

[15] Dellon ES Gonsalves N Hirano I Furuta GT Liacouras CA Katzka DA. ACG clinical guideline: Evidenced based approach to the diagnosis and management of esophageal eosinophilia and eosinophilic esophagitis (EoE). Am J Gastroenterol. (2013) 108:679–92. 10.1038/ajg.2013.71 23567357

[16] Lucendo AJ Arias Á Molina-Infante J. Efficacy of proton pump inhibitor drugs for inducing clinical and histologic remission in patients with symptomatic esophageal eosinophilia: a systematic review and meta-analysis. Clin Gastroenterol Hepatol. (2016) 14:13–22. 10.1016/j.cgh.2015.07.041 26247167

[17] Rea F Caldaro T Tambucci R Romeo EF Caloisi C Torroni F. Eosinophilic esophagitis: is it also a surgical disease? J Pediatr Surg. (2013) 48:304–8. 10.1016/j.jpedsurg.2012.11.006 23414856

[18] Schroeder S Capocelli KE Masterson JC Harris R Protheroe C Lee JJ. Effect of proton pump inhibitor on esophageal eosinophilia. J Pediatr Gastroenterol Nutr. (2013) 56:166–72. 10.1097/MPG.0b013e3182716b7a 23325438 PMC3552376

[19] Gutiérrez-Junquera C Fernández-Fernández S Cilleruelo ML Rayo A Echeverría L Quevedo S. High prevalence of response to proton-pump inhibitor treatment in children with esophageal eosinophilia. J Pediatr Gastroenterol Nutr. (2016) 62:704–10. 10.1097/MPG.0000000000001019 26513622

[20] Moawad FJ Schoepfer AM Safroneeva E Ally MR Chen YJ Maydonovitch CL. Eosinophilic oesophagitis and proton pump inhibitor-responsive oesophageal eosinophilia have similar clinical, endoscopic and histological findings. Aliment Pharmacol Ther. (2014) 39:603–8. 10.1111/apt.12636 24461332

[21] Vazquez-Elizondo G Ngamruengphong S Khrisna M Devault KR Talley NJ Achem SR. The outcome of patients with oesophageal eosinophilic infiltration after an eight-week trial of a proton pump inhibitor. Aliment Pharmacol Ther. (2013) 38:1312–9. 10.1111/apt.12513 24117619

[22] Moawad FJ Veerappan GR Dias JA Baker TP Maydonovitch CL Wong RKH. Randomized controlled trial comparing aerosolized swallowed fluticasone to esomeprazole for esophageal eosinophilia. Am J Gastroenterol. (2013) 108:366–72. 10.1038/ajg.2012.443 23399553

[23] Warners MJ van Rhijn BD Curvers WL Smout AJPM Bredenoord AJ. PPI-responsive esophageal eosinophilia cannot be distinguished from eosinophilic esophagitis by endoscopic signs. Eur J Gastroenterol Hepatol. (2015) 27:506–11. 10.1097/MEG.0000000000000331 25822858

[24] Sodikoff J Hirano I Proton pump inhibitor-responsive esophageal eosinophilia does not preclude food-responsive eosinophilic esophagitis. J Allergy Clin Immunol. (2016) 137:631–3. 10.1016/j.jaci.2015.07.008 26318073

[25] Lucendo AJ Arias Á González-Cervera J Olalla JM Molina-Infante J. Dual response to dietary/topical steroid and proton pump inhibitor therapy in adult patients with eosinophilic esophagitis. J Allergy Clin Immunol. (2016) 137:931–4. 10.1016/j.jaci.2015.07.033 26371836

[26] Dohil R Newbury RO Aceves S. Transient PPI responsive esophageal eosinophilia may be a clinical sub-phenotype of pediatric eosinophilic esophagitis. Dig Dis Sci. (2012) 57:1413–9. 10.1007/s10620-011-1991-5 22134787

[27] Gutiérrez-Junquera C Fernández-Fernández S Cilleruelo ML Rayo A Echeverría L Borrell B. Long-term treatment with proton pump inhibitors is effective in children with eosinophilic esophagitis. J Pediatr Gastroenterol Nutr. (2018) [Epub ahead of print]. 10.1097/MPG.0000000000001952 29509636

[28] Gómez-Torrijos E García-Rodríguez R Castro-Jiménez A Rodríguez-Sanchez J Méndez Díaz Y Molina-Infante J. The efficacy of step-down therapy in adult patients with proton pump inhibitor-responsive oesophageal eosinophilia. Aliment Pharmacol Ther. (2016) 43:534–40. 10.1111/apt.13496 26662868

[29] Molina-Infante J Rodriguez-Sanchez J Martinek J van Rhijn BD Krajciova J Rivas MD. Long-Term loss of response in proton pump inhibitor-responsive esophageal eosinophilia is uncommon and influenced by CYP2C19 genotype and rhinoconjunctivitis. Am J Gastroenterol. (2015) 110:1567–75. 10.1038/ajg.2015.314 26416193

[30] Dellon ES Speck O Woodward K Covey S Rusin S Gebhart JH. Markers of eosinophilic inflammation for diagnosis of eosinophilic esophagitis and proton pump inhibitor-responsive esophageal eosinophilia: a prospective study. Clin Gastroenterol Hepatol. (2014) 12:2015–22. 10.1016/j.cgh.2014.06.019 24993367 PMC4252508

[31] Wen T Stucke EM Grotjan TM Kemme KA Abonia JP Putnam PE. Molecular diagnosis of eosinophilic esophagitis by gene expression profiling. Gastroenterology (2013) 145:1289–99. 10.1053/j.gastro.2013.08.046 23978633 PMC4070519

[32] Wen T Rothenberg ME. Clinical applications of the eosinophilic esophagitis diagnostic panel. Front Med. (2017) 4:108. 10.3389/fmed.2017.00108 28770203 PMC5509802

[33] Wen T Dellon ES Moawad FJ Furuta GT Aceves SS Rothenberg ME. Transcriptome analysis of proton pump inhibitor-responsive esophageal eosinophilia reveals proton pump inhibitor-reversible allergic inflammation. J Allergy Clin Immunol. (2015) 35:187–97. 10.1016/j.jaci.2014.08.043 25441638 PMC4289084

[34] Shoda T Matsuda A Nomura I Okada N Orihara K Mikami H. Eosinophilic esophagitis versus proton pump inhibitor-responsive esophageal eosinophilia: transcriptome analysis. J Allergy Clin Immunol. (2017) 139:2010–2013. 10.1016/j.jaci.2016.11.028 28063872

[35] Molina-Infante J Rivas MD Hernandez-Alonso M Vinagre-Rodríguez G Mateos-Rodríguez JM Dueñas-Sadornil C. Proton pump inhibitor-responsive oesophageal eosinophilia correlates with downregulation of eotaxin-3 and Th2 cytokines overexpression. Aliment Pharmacol Ther. (2014) 40:955–65. 10.1111/apt.12914 25112708

[36] Park JY Zhang X Nguyen N Souza RF Spechler SJ Cheng E. Proton pump inhibitors decrease eotaxin-3 expression in the proximal esophagus of children with esophageal eosinophilia. PLoS ONE (2014) 9:101391. 10.1371/journal.pone.0101391 24988451 PMC4079672

[37] Lucendo AJ Molina-Infante J Arias Á von Arnim U Bredenoord AJ Bussmann C. Guidelines on eosinophilic esophagitis: evidence-based statements and recommendations for diagnosis and management in children and adults. United Eur Gastroenterol J. (2017) 5:335–58. 10.1177/2050640616689525 28507746 PMC5415218

[38] Reed CC Wolf WA Cotton CC Rusin S Perjar I Hollyfield J. Optimal histologic cutpoints for treatment response in patients with eosinophilic esophagitis: analysis of data from a prospective cohort study. Clin Gastroenterol Hepatol. (2018) 16:226–233. 10.1016/j.cgh.2017.09.046 28987502 PMC6582220

[39] Martin LJ Franciosi JP Collins MH Abonia JP Lee JJ Hommel KA. Pediatric Eosinophilic Esophagitis Symptom Scores (PEESS v2.0) identify histologic and molecular correlates of the key clinical features of disease. J Allergy Clin Immunol. (2015) 135:1519–28. 10.1016/j.jaci.2015.03.004 26051952 PMC4460579

[40] Safroneeva E Straumann A Coslovsky M Zwahlen M Kuehni C Panczak R. Symptoms have modest accuracy in detecting endoscopic and histologic remission in adults with eosinophilic esophagitis. Gastroenterology (2016) 150:581–90. 10.1053/j.gastro.2015.11.004 26584601 PMC6011000

[41] van Rhijn BD Weijenborg PW Verheij J van den Bergh Weerman MA Verseijden C van den Wijngaard RM Proton pump inhibitors partially restore mucosal integrity in patients with proton pump inhibitor-responsive esophageal eosinophilia but not eosinophilic esophagitis. Clin Gastroenterol Hepatol. (2014) 12:1815–23. 10.1016/j.cgh.2014.02.037 24657840

[42] Ishimura N Ishihara S Kinoshita Y. Sustained Acid Suppression by Potassium-Competitive Acid Blocker (P-CAB) May be an attractive treatment candidate for patients with eosinophilic esophagitis. Am. J. Gastroenterol. (2016) 111:1203–4. 10.1038/ajg.2016.167 27481423

[43] Zhang X Cheng E Huo X Yu C Zhang Q Pham TH. Omeprazole blocks STAT6 binding to the eotaxin-3 promoter in eosinophilic esophagitis cells. PLoS ONE (2012) 7:50037. 10.1371/journal.pone.0050037 23185525 PMC3503709

[44] Cheng E Zhang X Wilson KS Wang DH Park JY Huo X. JAK-STAT6 pathway inhibitors block eotaxin-3 secretion by epithelial cells and fibroblasts from esophageal eosinophilia patients: promising agents to improve inflammation and prevent fibrosis in EoE. PLoS ONE (2016) 11:0157376. 10.1371/journal.pone.0157376 27310888 PMC4911010

[45] Soller L Mill C Avinashi V Teoh T Chan ES. Development of anaphylactic cow's milk allergy following cow's milk elimination for eosinophilic esophagitis in a teenager. J Allergy Clin Immunol Pract. (2017) 5:1413–4. 10.1016/j.jaip.2017.02.021 28389301

[46] Echeverría-Zudaire LÁ Fernández-Fernández S Rayo-Fernández A Muñóz-Archidona C Checa-Rodriguez R. Primary eosinophilic gastrointestinal disorders in children who have received food oral immunotherapy. Allergol. Immunopathol. (2016) 44:531–6. 10.1016/j.aller.2016.05.002 27592279

[47] Yadlapati R Kahrilas PJ. The “dangers” of chronic proton pump inhibitor use. J Allergy Clin Immunol. (2018) 141:79–81. 10.1016/j.jaci.2017.06.017 28729001

[48] Attwood SE Ell C Galmiche JP Fiocca R Hatlebakk JG Hasselgren B. Long-term safety of proton pump inhibitor therapy assessed under controlled, randomised clinical trial conditions: data from the SOPRAN and LOTUS studies. Aliment Pharmacol Ther. (2015) 41:1162–74. 10.1111/apt.13194 25858519

[49] Tjon JA Pe M Soscia J Mahant S. Efficacy and safety of proton pump inhibitors in the management of pediatric gastroesophageal reflux disease. Pharmacotherapy (2013) 33:956–71. 10.1002/phar.1299 23712734

[50] Writing Committee for the American Lung Association Asthma Clinical Research Centers Holbrook JT Wise RA Gold BD Blake K Brown ED. Lansoprazole for children with poorly controlled asthma: a randomized controlled trial. JAMA (2012) 307:373–81. 10.1001/jama.2011.2035 22274684 PMC4153372

[51] Liacouras CA Spergel J Gober LM. Eosinophilic esophagitis: clinical presentation in children. Gastroenterol Clin North Am. (2014) 43:219–29. 10.1016/j.gtc.2014.02.012 24813511

